# The impact of reproductive life on breast cancer risk in women with family history or BRCA mutation

**DOI:** 10.18632/oncotarget.13423

**Published:** 2016-11-17

**Authors:** Angela Toss, Giovanni Grandi, Angelo Cagnacci, Luigi Marcheselli, Silvia Pavesi, Elisabetta De Matteis, Elisabetta Razzaboni, Chiara Tomasello, Stefano Cascinu, Laura Cortesi

**Affiliations:** ^1^ Department of Oncology and Haematology, Azienda Ospedaliero-Universitaria Policlinico, University of Modena and Reggio Emilia, Modena, Italy; ^2^ Department of Obstetrics Gynecology and Pediatrics, Obstetrics and Gynecology Unit, Azienda Ospedaliero-Universitaria Policlinico, University of Modena and Reggio Emilia, Modena, Italy; ^3^ Department of Oncology, Vito Fazzi Hospital, Lecce, Italy

**Keywords:** breast cancer, family history, BRCA, reproductive factors, combined hormonal contraceptive

## Abstract

Reproductive history and exogenous hormonal exposures are acknowledged risk factors for breast cancer in the general population. In women at increased breast cancer risk for genetic predisposition or positive family history, data regarding these risk factors are limited or conflicting, and recommendations for these categories are unclear. We evaluated the characteristics of reproductive life in 2522 women at increased genetic or familial breast cancer risk attending our Family Cancer Center. Breast cancers in BRCA mutation carriers were more likely to be hormone receptor negative, diagnosed at 35 years or before and multiple during the lifetime than tumors in women at increased familial risk, while the distribution of invasive cancers and HER2 positive tumors was similar in the different risk groups. At least one full-term pregnancy (HR 0.27; 95% CI 0.12–0.58; *p* = 0.001), breastfeeding either less (HR 0.24; 95% CI 0.09–0.66; *p* = 0.005) or more (HR 0.25; 95% IC 0.08–0.82; *p* = 0.022) than one year and late age at menopause (HR 0.10; 95% CI 0.01–0.82; *p* = 0.033) showed to be protective factors in BRCA mutation carriers, while in women at increased familial risk early age at first full-term pregnancy (HR 0.62; 95% IC 0.38–0.99; *p* = 0.048) and late menarche (HR 0.61; 95% CI 0.42–0.85; *p* = 0.004) showed to be the main protective factors. Finally, for the entire population, combined hormonal contraceptives demonstrated to do not increase breast cancer risk. The results of our study suggest that women at high familial risk and mutation carries develop tumors with different clinical-pathological characteristics and, consequently, are influenced by different protective and risk factors.

## INTRODUCTION

Breast cancer (BC) represents the most common form of cancer and the leading cause of cancer death among women worldwide, with an estimated 1.7 million cases and 521,900 deaths in 2012. BC alone accounts for 25% of all cancer cases and 15% of all cancer deaths among females [1].

In the general population, acknowledged risk factors for BC are aging, white race, reproductive history (i.e. early menarche, late menopause, nulliparity, late age at first birth, no breastfeeding), exposures (i.e. exogenous hormones like combined hormonal contraceptives (CHCs) or hormone replacement therapy), alcohol, tobacco smoking, overweight and obesity, lack of exercise, dense breast tissue, previous chest radiation, precancerous lesions, personal history of BC, family history and mutations in BC predisposition genes (BRCA1, BRCA2, PALB2, PTEN, CHEK2, etc.) [2–13].

These risk factors have also been investigated in the high-risk population of BRCA mutation carriers in a variety of studies. Particularly, a recent meta-analysis [14] showed that, in BRCA1-mutation carriers, the only factor that both provided sufficient evidence from the grading analysis and for which a meta-analysis could be derived, was the protective effect of late age at first birth. This is inconsistent with the effect in the general population. On the other hand, no reproductive factor was unequivocally associated with risk modification in BRCA2-mutation carriers. Interestingly, meta-analysis of case-control studies of oral contraceptives use in mutation carriers resulted in no association with BC risk. In contrast, meta-analysis of prospective cohort studies indicated an increase in BC risk among mutation carriers. Therefore, different study designs revealed conflicting results and most of the associations were deficient in one or more ways. Nevertheless, the authors concluded that, despite the limitations of the data available to address which factors are associated with cancer risk, many of the associations are largely consistent with the effects that would be predicted in the general population [14]. For what concerns women at increased familial risk with unknown mutation status, the data available are even more limited and conflicting.

In conclusion, it results unclear the extent to which these modifiers of risk influence BC risk in BRCA mutation carriers and in women with a family history of BC. Nonetheless, an accurate risk assessment in these high-risk populations remains fundamental, in order to establish personalized strategies for risk control (i.e. surveillance, chemoprevention [15] and prophylactic surgery [16]). At this aim, reproductive factors were investigated in a population of Italian women attending the Modena Family Cancer Clinic (MFCC), comparing women classified at different levels of increased BC risk.

## RESULTS

We collected the main reproductive factors through the revision of the medical records of 2527 women attending the MFCC between May 2010 and January 2016. One woman with a MLH1 mutation and 4 women who underwent bilateral prophylactic mastectomy have been subsequently excluded, thus 2522 women were finally analyzed. Characteristics of the entire population divided by subgroups and the distribution of reproductive variables are presented in Table 1. The characteristics of reproductive life were homogeneous among the three groups, except for nulliparity that was significantly more frequent in the mutation carriers (*p <* 0.001).

**Table 1:** Characteristics of the entire population by divided subgroups and the distribution of reproductive variables

	VHR Group	HR Group	IR Group	*p*-value
**N° of women**	113	1541	868	
**Mean age (SD)**	51 (14)	52 (13)	52 (12)	0.634
**Pregnancy (%)** (2094 women)				
Yes	59 (69)	1100 (86)	618 (85)	< 0.001
No	26 (31)	180 (14)	111 (15)	
**N° of pregnancies (%)** (1777 women)				
1	18 (30)	493 (45)	276 (45)	0.271
2	34 (58)	509 (46)	288 (47)	
3+	7 (12)	98 (9)	54 (9)	
**Age at first full term pregnancy (%)** (1725 women)				
≤ 30 years	42 (78)	829 (77)	446 (74)	0.304
> 30 years	12 (22)	241 (23)	155 (26)	
**Breastfeeding (%)** (926 women)				
Pregnancy without breastfeeding	7 (17)	104 (19)	49 (15)	0.300
1–12 months	20 (49)	295 (53)	171 (51)	
> 12 months	14 (34)	154 (28)	112 (34)	
**Menarche (%)** (2522 women)				
≤ 12 years	73 (65)	893 (58)	493 (57)	0.285
> 12 years	40 (35)	648 (42)	375 (43)	
**Menopause (%)** (359 women)				
≤ 50 years	8 (53)	144 (59)	56 (57)	0.868
> 50 years	7 (47)	101 (41)	43 (43)	
**Abortion (%)** (1078 women)				
Yes	13 (25)	171 (29)	125 (29)	0.762
No	40 (75)	428 (71)	301 (71)	
**CHC use (%)** (1467 women)				
Present/Previous	41 (58)	604 (69)	373 (71)	0.060
Never	30 (42)	270 (31)	149 (29)	
**CHC Duration (%)** (1457 women)				
≤ 10 years/no use	65 (92)	739 (86)	446 (87)	0.139
> 10 years	6 (8)	135 (15)	66 (13)	

Statistical test: one-way ANOVA for age; for categorical variables chi2 test or Fischer's exact test, when appropriate.

Abbreviations: VHR = Very High Risk, HR = High Risk, IR = Intermediate Risk, SD = Standard Deviation.

The mean age ± SD of the entire population at the end of the period was 52 ± 13 years, ranging from 17 years to 92 years. Among these women, 113 (5%) had a pathogenic mutation in one of the BRCA genes (VHR Group). The mean age for the mutation carriers at the end of the period was 51 ± 14, years and they were 64 BRCA1 and 49 BRCA2. In addition, 1541 (61%) were at High Risk (HR) with a mean age of 52 ± 13 years and 868 (34%) women were at Intermediate Risk (IR) with a mean age of 52 ± 12 years, according to the Modena criteria. Fourteen women with a BRCA variant of unknown significance (VUS) have been classified according to their family history (2 at Intermediate Risk and 12 at High Risk).

**Figure 1:**
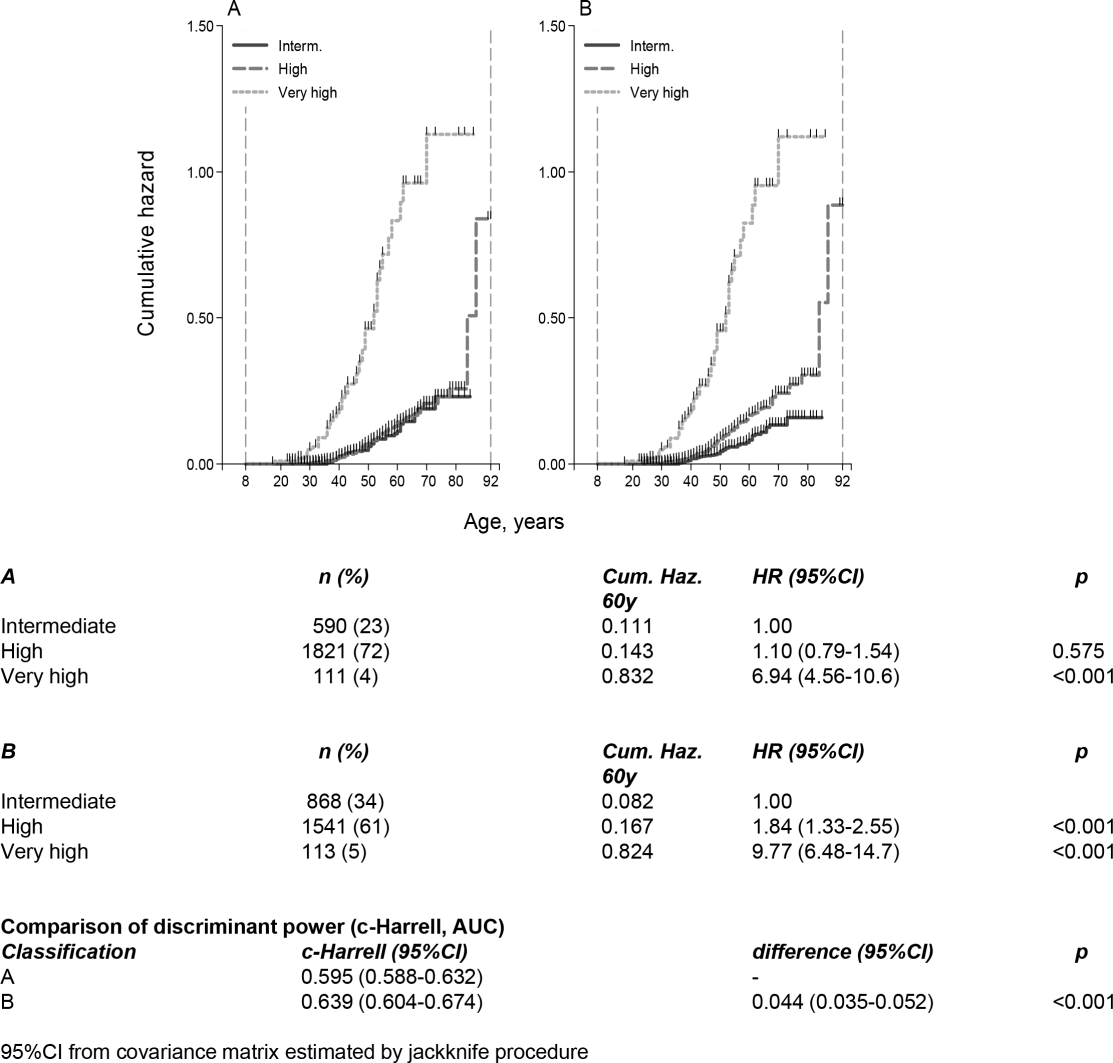
(**A**) Cumulative Nelson-Aalen hazard by risk groups according to the combination of Tyrer-Cuzick model and the Modena Criteria; (**B**) Cumulative Nelson-Aalen hazard by risk groups according to the Modena criteria. Comparison of discriminant power (c-Harrell, AUC).

Overall, 255 women developed BC during their lifetime, at a median age of 48 years (SD 11, range 18–87). One hundred and sixty-three of these women developed BC before the first access at the MCFF. The Modena Criteria correctly identified 3 different categories at increasing cumulative BC risk (*p* < 0.001) (Figure 1).

The characteristics of tumors developed by each group are summarized in Table 2. Particularly, BCs in BRCA carriers were more likely to be hormone receptor negative (*p* = 0.024), diagnosed at 35 years or before (*p* = 0.002) and multiple during the lifetime (*p* = 0.017). Consequently, age at diagnosis was significantly lower in VHR compared to HR and IR (44 vs. 49 vs. 50 years, respectively) (*p* = 0.007). On the contrary, the distribution of invasive BC (*p* = 0.338) and HER2 positive tumors (*p* = 0.545) was similar in the three groups of patients. Nevertheless, it should be noted that BRCA1-related BC were significantly more likely to be hormone receptor negative than BRCA2-related tumors (*p* = 0.036). Moreover, somewhat surprisingly, we found a higher rate of HER2-negative tumors than that seen in the general population for all the three groups at increasing risk (respectively, 87%, 91% and 96%).

**Table 2:** The characteristics of tumors developed by each risk group

	VHR Group	HR Group	IR Group	*p*-value
**N° of BC** (255 patients)	45 (40)	160 (10)	50 (6)	< 0.001
**Mean age (SD)**	44 (11)	49 (11)	50 (10)	0.007
**HER2 status (%)** (143 patients)				
Positive	1 (4)	8 (9)	4 (15)	0.545
Negative	24 (96)	80 (91)	26 (87)	
**Hormone Receptor Status (%)** (174 patients)				
Positive	21 (60)	88 (83)	25 (76)	0.024
Negative	14 (40)	18 (17)	8 (24)	
**Age at diagnosis (%)** (248 patients)				
≤ 35 years	9 (20)	16 (10)	0	0.002
> 35 years	35 (80)	139 (90)	49 (100)	
**Invasive Carcinoma (%)** (225 patients)				
Yes	34 (85)	108 (78)	33 (72)	0.338
No	6 (15)	31 (22)	13 (28)	
**Multiple Carcinoma in the lifetime (%)** (247 patients)				
Yes	10 (23)	11 (7)	4 (8)	0.017
No	34 (77)	144 (93)	44 (92)	

Statistical test: one-way ANOVA for age; for categorical variables chi2 test or Fischer's exact test, when appropriate.

Abbreviations: VHR = Very High Risk, HR = High Risk, IR = Intermediate Risk, BC = Breast Cancer, SD = Standard Deviation.

The cumulative risks at 60 years according to the characteristics of reproductive life and CHCs use for each risk category are presented in Table 3.

**Table 3:** The cumulative risks at 60 years according to the characteristics of reproductive life and oral contraception for each risk category

	Hazard Ratio	95% CI	*p*-value
**Pregnancy, yes**			
VHR	0.27	0.12–0.58	0.001
HR	0.94	0.43–2.02	0.869
IR	1.00	0.31–3.29	0.999
**N° of pregnancies, 1**			
VHR	0.31	0.12–0.85	0.022
HR	0.84	0.37–1.87	0.662
IR	0.75	0.22–2.63	0.650
**N° of pregnancies, 2**			
VHR	0.29	0.12–0.66	0.003
HR	0.99	0.45–2.18	0.975
IR	1.09	0.32–3.69	0.885
**N° of pregnancies, 3**			
VHR	0.08	0.01–0.67	0.019
HR	1.17	0.46–2.99	0.740
IR	1.80	0.45–7.20	0.407
**Age at first full term pregnancy, ≤ 30 years**			
VHR	0.49	0.19–1.26	0.138
HR	0.62	0.38–0.99	0.048
IR	0.61	0.29–1.32	0.213
**Breastfeeding, 1–12 months**			
VHR	0.24	0.09–0.66	0.005
HR	1.12	0.49–2.58	0.784
IR	1.49	0.42–5.22	0.536
**Breastfeeding, > 12 months**			
VHR	0.25	0.08–0.82	0.022
HR	1.91	0.79–4.61	0.151
IR	1.86	0.49–7.03	0.357
**Menarche, > 12 years**			
VHR	1.23	0.67–2.25	0.504
HR	0.61	0.42–0.85	0.004
IR	0.93	0.52–1.63	0.792
**Menopause, > 50 years**			
VHR	0.10	0.01–0.82	0.033
HR	0.57	0.25–1.32	0.192
IR	0.55	0.10–3.00	0.489
**Abortion, yes**			
VHR	0.61	0.20–1.85	0.384
HR	0.99	0.59–1.68	0.987
IR	2.63	1.18–5.88	0.018
**CHC use, Current/Past**			
VHR	1.45	0.65–3.25	0.361
HR	1.03	0.65–1.63	0.901
IR	0.76	0.36–1.58	0.459
**CHC Duration, > 10 years**			
VHR	0.78	0.18–3.34	0.742
HR	1.17	0.65–2.13	0.600
IR	0.56	0.13–2.34	0.424

Abbreviations: 95% CI = 95% Confidence Interval, VHR = Very High Risk, HR = High Risk, IR = Intermediate Risk.

### Effect of pregnancy

Among BRCA mutation carriers, women with at least one full-term pregnancy compared with nulliparous women had a lower risk of BC (*p* = 0.001). In order to avoid the potential bias due to BC detected before pregnancy, in this analysis we included women that developed cancer before getting pregnant in the group of nulliparous. Notably, among parous women, an increasing number of full-term pregnancies seem to be associated with a decrease in BC risk (HR for 1 pregnancy = 0.31, HR for 2 pregnancies = 0.29, HR for 3 or more pregnancies = 0.08). Interestingly, only in HR group, age at first live birth ≤ 30 showed to significantly reduce risk for BC (*p* = 0.048).

### Effect of breastfeeding

We observed no association between ever having breast-fed and BC risk, among women at increased familial risk (HR and IR groups). In these two groups, there was also no statistically significant association between duration of breastfeeding (more or less than 12 months) and BC risk. Interestingly, only in VHR group, breastfeeding either less (*p* = 0.005) or more (*p* = 0.022) than one year showed to significantly reduce the risk for BC.

### Effect of miscarriage or abortion

We found no association between having a miscarriage or abortion and the risk of BC for the VHR group (*p* = 0.384) and for the HR group (*p* = 0.987). Conversely, in the IR group a miscarriage or an abortion seems to have increased the risk of BC (*p* = 0.018).

### Effect of menarche and menopause

In HR women, a late age at menarche (> 12 years) showed to significantly reduce risk for BC (*p* = 0.004). For the analysis of menopausal age, we’ve considered only women diagnosed after their menopause. Interestingly, late menopause (> 50 years) seems to be even protective in BRCA mutation carriers (*p* = 0.033).

### Effect of use of combined hormonal contraceptives (CHC)

The current or past use of CHC did not show to significantly modify risk for BC in all the risk groups. Particularly, BC risk was not influenced by CHC duration of assumption and also an assumption longer than 10 years did not increase BC risk in all the groups.

## DISCUSSION

Reproductive history and exogenous hormonal exposures are acknowledged risk factors for BC in general population [2–13]. Nevertheless, in women at increased BC risk for genetic predisposition or positive family history, data regarding these risk factors are limited or conflicting, and recommendations for these categories are unclear. On these bases, we decided to carry out an observational retrospective study among women at increased genetic or familial BC risk attending our Institution, one of the largest and most specialized Family Cancer Clinic in Italy. Characteristics of reproductive life have been evaluated in 2522 women according to their risk profile. Women have been classified according to the Modena Criteria [17, 18], which demonstrated to be able to correctly stratify the risk in women with a family history of breast cancer.

In the present study, we found that at least one full-term pregnancy represents a protective factor for BC in VHR group, and an increasing number of full-term pregnancies is associated with a proportional decrease in BC risk. On the other hand, age at first full-term pregnancy ≤ 30 years showed to be a protective factor only in women at high familial risk for BC (HR Group). In previous literature, age at first full-term pregnancy demonstrated to be a powerful denominator of future BC risk in the general population, as it determines the length of time the undifferentiated breast tissue remains particularly vulnerable [19–21]. Interestingly, it has been reported that older age at first full-term pregnancy has also an impact on the molecular subtypes of BC and, it has been associated with increased risk for luminal BC and lobular histotype [4, 22–24]. In our BRCA mutated subjects, the association between early first full-term pregnancy and decreased BC risk was not significant, as also reported in previous research [14]. This finding could be explained by the fact that BRCA carriers tend to develop more frequently hormone receptor negative tumors, while age at first full-term pregnancy seems to modify mostly the incidence of hormone receptor positive tumors [4, 22–24].

After pregnancy, an extended period of breastfeeding contributes to the functional ripening of the glandular tissue. On these bases, the duration of breastfeeding is believed to have an influence on BC risk. Particularly, the Collaborative Group on Hormonal Factors in Breast Cancer found that the RR for BC decreased by 4.3% (*p* < 0.0001) for every 12 months of breastfeeding [3]. Interestingly, a recent workshop on postpartum remodeling, lactation, and BC risk held by the National Cancer Institute suggested a particular protection against triple-negative, basal-like, and BRCA1 mutation-associated BC [25]. Moreover, two recent meta-analyses confirmed the protective effect of ever breastfeeding against hormone receptor-negative BC, which are more common in younger women and BRCA mutation carriers [24, 26]. Our results confirm these previous findings, since we found a protective effect of breastfeeding only in BRCA mutation carriers that, also in our sample, are more likely to be affected by hormone receptor negative BC.

As regards the effects of previous miscarriages/abortions, a collaborative reanalysis including 83,000 women with BC showed that miscarriages and abortions do not increase BC risk [27] and the same was found In Chinese women, specifically for abortion [28]. On the other hand, in BRCA mutation carriers data on the association between incomplete pregnancies and a higher BC risk are conflicting [29, 30]. Our results showed that miscarriages and abortions increase the risk of BC only in the IR group. These findings should be confirmed in larger samples and with the evaluation of other cofounders such as total months of these pregnancies and age at miscarriage/abortion.

In 2012, a meta-analysis including 118,964 women with invasive BC and 306,091 without the disease, showed that BC risk increases by a factor of 1.050 (95% CI 1.044–1.057) for every year younger at menarche, and independently by a smaller amount (1.029; 95% CI 1.025–1.032), for every year older at menopause. Particularly, exposure to endogenous ovarian hormones are more relevant for estrogen receptor positive disease than for estrogen receptor negative disease and for lobular than for ductal tumors [4, 5]. In our study, a late age at menarche (> 12 years) showed to significantly reduce risk for BC in HR women according to previous research. Conversely, in our BRCA mutation carriers, menarcheal age does not influence BC risk and, interestingly, late menopause (> 50 years) seems to be even protective. Nevertheless, it should be noted that we excluded from the analysis all the patients that developed BC before menopause, therefore most of VHR women with BC in our sample, decreasing the statistical power of this analysis. Interestingly, among women considered for this analysis, 50% of women affected by BC went through menopause after bilateral (prophylactic or therapeutic) oophorectomy before the BC development, while only 22.2% of women who did not develop BC underwent bilateral oophorectomy after the natural menopause. However, it should be noted that the tumors developed by these women were triple-negative BC. Overall, these findings confirm the fact that endogenous ovarian hormones are more relevant for estrogen receptor positive disease.

Several studies have found that women using oral contraceptives have a slightly greater risk of BC than women who have never used them. This risk seems to go back to normal over time once the pills are stopped. Nevertheless, these studies mostly refer to CHC used before 1975 and may be the result of exposure to higher dosages of estrogens [31, 32]. Moreover, these results have been confuted by other studies, showing no association between CHC and BC risk. Therefore, different studies revealed conflicting results in both general population and women at increased familial or genetic BC risk [14]. In our study, the current or past use of CHC did not show to significantly modify the risk for BC in all the risk groups, even if the duration of assumption was longer than 10 years. One possible implication of these results, along with data available in literature, is that chemoprevention for ovarian cancer with CHC may be encouraged among BRCA mutation carriers.

One of the main limitations of our study consists in the lack of data, because several medical records have not been entirely filled. As regards tumor characteristics, the immune-histochemical features were available only for women with invasive carcinomas, diagnosed or treated in the province of Modena in the last 20 years, or for women who provided their past documentation. Moreover, the systematic evaluation of HER2 status became available in our Institution only in 2006, when Trastuzumab has been approved in the adjuvant setting. Another limitation of our study is the non-availability of information regarding start age for CHC, breastfeeding and abortion, which showed in previous literature to play a role in the modulation of BC risk.

Somewhat unexpectedly, we found different risk and protective factors between HR and IR women (age at menarche, age at first full term pregnancy and miscarriages/abortions). These results may have three possible explanations. Firstly, the IR group is smaller and with fewer events than the HR group, and this might have affected the statistical power of the analyses. Secondly, we do not have any information regarding the presence of other possible pathogenic mutations in other susceptibility genes in this population of women. It is likely that a part of these women with positive family history, especially in the HR group, may carry an inherited mutation other than BRCA, which may have contributed to modify their risk of developing BC, irrespectively of the characteristics of reproductive life. Finally, women belonging to the IR group might have a lower risk perception compared to HR risk women, because of their slighter familiarity and less intensive surveillance program. Therefore, IR women might have worked less hard in order to change their modifiable risk factors, such as alcohol assumption, tobacco smoking, dietary habits and physical exercise. Overall, these missing genetic and lifestyle factors represent possible confounders in our analysis.

Overall, in our study at least one full-term pregnancy, breastfeeding and late age (> 50 years) at menopause represent the principal protective factors in BRCA mutation carriers, while in women at increased familial risk early age at first full-term pregnancy and late menarche represent the principal protective factors. These data are relevant in particular for BRCA mutation carriers, which in our sample got fewer pregnancies than other women, maybe because of the concern of mutation inheritance. These results confirm that pregnancies and the reproductive lifespan play crucial roles in the definition of BC risk in women at increased familial and genetic risk. Moreover, women at high familial risk and mutation carries develop tumors with different clinical-pathological features and, consequently, are influenced by different protective and risk factors. Nevertheless, it is noteworthy that in our sample as well as in literature, hormone receptor negative tumors are mainly associated with BRCA1-mutation carriers, while patients with a BRCA2 mutation seem to develop tumors with histopathological profile more similar to that of tumors affecting the general population [33, 34]. Therefore, further investigations into the correlations among clinical-pathological features and reproductive factors in the two different subgroups of BRCA-mutation carriers are needed.

## MATERIALS AND METHODS

Since 1995, the MFCC classifies women with a family history of breast and/or ovarian cancer according to the Modena criteria [17, 18] and, more recently, by the Tyrer-Cuzick model [35] and provide personalized surveillance programs.

The Modena criteria used to classify families are the following:

Criterion 1: At least three relatives with BC in two different generations, regardless of family structure. Alternatively, two relatives with BC and one with OC or one with BC and two with OC in two different generations.
Criterion 2: At least two family members affected by BC with a first-degree relationship. Alternatively, one relative with BC and one with OC. In the case of male interposition, a relationship of different degree (second or third) is allowed.
Criterion 3: At least one BC diagnosed before 40 years of age or bilateral.

According to these criteria, families are divided into main clusters (Table 4): Hereditary Breast (and Ovarian) Cancer (HBC/HBOC), Suspected Hereditary Breast (and Ovarian) Cancer (SHBC/SHBOC), Familial Breast (and Ovarian) Cancer (FBC/FBOC), Suspected Familial Breast (and Ovarian) Cancer (SFBC/SFBOC), Strongly Suspected Familial Breast (and Ovarian) Cancer (SFBC+/SFBOC+).

**Table 4:** The modena criteria for classification of hereditary and familial predisposition to breast and ovarian cancer

CRITERION 1 three relatives with BC/OC in two different generations	CRITERION 2 first-degree relationship	CRITERION 3 BC diagnosed before 40 years or bilateral	CLASSIFICATION
X	X	X	**HBC/HBOC**
X	X		**SHBC/SHBOC**
X		X	
X			**FBC/FBOC**
	X	X	**SFBC+/SFBOC+**
	X		**SFBC/SFBOC**
		X	

Together with these basic clusters, some further criteria have been added (Table 5): Early Onset Breast Cancer (EOBC), Breast and Ovarian Cancer (BOC), Male Breast Cancer (MBC), Hereditary Ovarian Cancer (HOC), and Familial Ovarian Cancer (FOC). In the absence of any criteria, the cases are considered as Sporadic Breast or Ovarian Cancer (SpBC/SpOC).

**Table 5:** The additional modena criteria for classification of hereditary and familial predisposition to breast and ovarian cancer

ADDITIONAL CRITERIA	CLASSIFICATION
BC diagnosed ≤ 35 years	**EOBC**
BC and OC in the same woman	**BOC**
Male breast cancer	**MBC**
≥ 2 first-degree relatives diagnosed with OC	**HOC**
≥ 2 relatives diagnosed with OC, not HOC	**FOC**
BC/OC without any of the other criteria	**SpBC/SpOC**

The characteristics of reproductive life were obtained through the revision of the medical records of 2527 women that started their surveillance program at the MFCC between May 2010 and January 2016. Particularly, we evaluated number of pregnancies, age at first full-term pregnancy, breastfeeding, abortions, menarche, menopause, and use of combined hormonal contraceptives (CHC). Also women with a previous diagnosis of breast cancer are admitted to the Center and have been included in the present study. The aim of our study was to analyze the impact of reproductive history and exogenous hormonal exposures in the development of BC in a population of BRCA mutation carriers and in women with family history of breast and/or ovarian cancer.

At first, women were divided according to a combination of the Modena Criteria and the Tyrer-Cuzick model in the following three groups:

### Very high risk (VHR)

women with a recognized BRCA1/2 mutation at the genetic testing.

### High risk (HR)

women not tested for BRCA genes or with a non-informative result, with a 3-fold increased lifetime risk of developing BC according to the Tyrer-Cuzick model or belonging to HBC/HBOC, SHBC/SHBOC, BOC, HOC, and EOBC families according to the Modena criteria.

### Intermediate risk (IR)

women not tested for BRCA genes or with a non-informative result, not belonging to the High Risk Group, with a 2-fold increased lifetime risk of developing BC according to the Tyrer-Cuzick model or belonging to FBC/FBOC, SFBC/SFBOC, SFBC+/SFBOC+ and MBC families according to the Modena criteria.

Women already diagnosed with BC at the time of the first access were classified according to the Modena Criteria, since the Tyrer-Cuzick model should not be applied to affected women.

Interestingly, the combined classification did not show to correctly stratify the cumulative risk at 60 years of these women (Figure 1A) and HR women demonstrated to have the same cumulative risk of IR women. For this reason, we subdivided our population exclusively according to the Modena Criteria in the following groups (Figure 1B).

### Very high risk (VHR)

women with a recognized BRCA1/2 mutation at the genetic testing.

### High risk (HR)

women not tested for BRCA genes or with a non-informative result, belonging to HBC/HBOC, SHBC/SHBOC, BOC, HOC, and EOBC families.

### Intermediate risk (IR)

women not tested for BRCA genes or with a non-informative result, belonging to FBC/FBOC, SFBC/SFBOC, SFBC+/SFBOC+ and MBC families.

Notably, women with a BRCA variant of unknown significance (VUS) have been classified according to their family history, as current Guidelines recommend. One woman with a MLH1 mutation and 3 women who underwent bilateral prophylactic mastectomy have been excluded from the analyses.

### Statistical methods

Since the median age of these women was quite low, we decided to evaluate the cumulative risk at 60 years according to the characteristics of their reproductive life. Analyses were conducted separately for subjects with mutations and women with a family history of BC according to the three risk categories. Unknown data (data not provided by women's medical records) has been excluded from the analyses.

In the descriptive analysis, the continuous variables were summarized by means of mean and standard deviation (SD), and the categorical variables were reported as absolute and percent values. Distributions of continuous covariates by group were compared using one-way ANOVA (analysis of variance). The comparison of categorical variables between groups was performed using the Chi2 test or Fisher's exact test, when appropriate. The cumulative risk to develop BC was calculated from the age of menarche to the age of diagnosis of BC, or age of the last control for censored patients. The cumulative hazard rate was calculated and graphically reported by means of Nelson-Aalen estimator [36]. The cumulative hazard rates comparison between groups were performed using the logrank test. The effect of covariates was reported as hazard ratio (HR) with 95% confidence interval (CI 95%), estimated from the Cox proportional hazard regression [37]. All the reported test were two-sided, and tests with conventional *p*-value < 0.05 were considered significant.

## References

[1] Torre LA, Bray F, Siegel RL, Ferlay J, Lortet-Tieulent J, Jemal A (2015). Global cancer statistics, 2012. CA Cancer J Clin.

[2] Ewertz M, Duffy SW, Adami HO, E Kvåle G Lund, Meirik O, Mellemgaard A, Soini I, Tulinius H (1990). Age at first birth, parity and risk of breast cancer: a meta-analysis of 8 studies from the Nordic countries. Int J Cancer.

[3] Collaborative Group on Hormonal Factors in Breast Cancer (2002). Breast cancer and breastfeeding: collaborative reanalysis of individual data from 47 epidemiological studies in 30 countries, including 50302 women with breast cancer and 96973 women without the disease. Lancet.

[4] Ritte R, Tikk K, Lukanova A, Tjønneland A, Olsen A, Overvad K, Dossus L, Fournier A, Clavel-Chapelon F, Grote V, Boeing H, Aleksandrova K, Trichopoulou A (2013). Reproductive factors and risk of hormone receptor positive and negative breast cancer: a cohort study. BMC Cancer.

[5] Collaborative Group on Hormonal Factors in Breast Cancer (2012). Menarche, menopause, and breast cancer risk: individual participant meta-analysis, including 118 964 women with breast cancer from 117 epidemiological studies. Lancet Oncol.

[6] Gierisch JM, Coeytaux RR, Urrutia RP, Havrilesky LJ, Moorman PG, Lowery WJ, Dinan M, McBroom AJ, Hasselblad V, Sanders GD, Myers ER (2013). Oral contraceptive use and risk of breast, cervical, colorectal, and endometrial cancers: a systematic review. Cancer Epidemiol Biomarkers Prev.

[7] Beral V, Million Women Study Collaborators (2003). Breast cancer and hormone-replacement therapy in the Million Women Study. Lancet.

[8] Collaborative Group on Hormonal Factors in Breast Cancer (2001). Familial breast cancer: collaborative reanalysis of individual data from 52 epidemiological studies including 58,209 women with breast cancer and 101,986 women without the disease. Lancet.

[9] Metcalfe K, Lubinski J, Lynch HT, Ghadirian P, Foulkes WD, Kim-Sing C, Neuhausen S, Tung N, Rosen B, Gronwald J, Ainsworth P, Sweet K, Eisen A (2010). Family history of cancer and cancer risks in women with BRCA1 or BRCA2 mutations. J Natl Cancer Inst.

[10] Zhang B, Beeghly-Fadiel A, Long J, Zheng W Genetic variants associated with breast-cancer risk: comprehensive research synopsis, meta-analysis, and epidemiological evidence. Lancet Oncol.

[11] Hartmann LC, Sellers TA, Frost MH, Lingle WL, Degnim AC, Ghosh K, Vierkant RA, Maloney SD, Pankratz VS, Hillman DW, Suman VJ, Johnson J, Blake C (2005). Benign breast disease and the risk of breast cancer. N Engl J Med.

[12] Mellemkjaer L, Friis S, Olsen JH, Scélo G, Hemminki K, Tracey E, Andersen A, Brewster DH, Pukkala E, McBride ML, Kliewer EV, Tonita JM, Kee-Seng C (2006). Risk of second cancer among women with breast cancer. Int J Cancer.

[13] Dossus L, Boutron-Ruault MC, Kaaks R, Gram IT, Vilier A, Fervers B, Manjer J, Tjonneland A, Olsen A, Overvad K, Chang-Claude J, Boeing H, Steffen A (2014). Active and passive cigarette smoking and breast cancer risk: results from the EPIC cohort. Int J Cancer.

[14] Friebel TM, Domchek SM, Rebbeck TR (2014). Modifiers of cancer risk in BRCA1 and BRCA2 mutation carriers: systematic review and meta-analysis. J Natl Cancer Inst.

[15] Razzaboni E, Toss A, Cortesi L, Marchi I, Sebastiani F, De Matteis E, Federico M (2013). Acceptability and adherence in a chemoprevention trial among women at increased risk for breast cancer attending the Modena Familial Breast and Ovarian Cancer Center (Italy). Breast J.

[16] Cortesi L, Razzaboni E, Toss A, De Matteis E, Marchi I, Medici V, Tazzioli G, Andreotti A, De Santis G, Pignatti M, Federico M (2014). A rapid genetic counselling and testing in newly diagnosed breast cancer is associated with high rate of risk-reducing mastectomy in BRCA1/2-positive Italian women. Ann Oncol.

[17] Federico M, Maiorana A, Mangone L, Turchetti D, Canossi B, Cortesi L, Romagnoli R, Silingardi V (1999). Identification of families with hereditary breast and ovarian cancer for clinical and mammographic surveillance: the Modena Study Group proposal. Breast Cancer Res Treat.

[18] Cortesi L, Turchetti D, Marchi I, Fracca A, Canossi B, Rachele B, Silvia R, Rita PA, Pietro T, Massimo F (2006). Breast cancer screening in women at increased risk according to different family histories: an update of the Modena Study Group experience. BMC Cancer.

[19] Hunt SC, Williams RR, Skolnick MH, Lyon JL, Smart CR (1980). Breast cancer and reproductive history from genealogical data. J Natl Cancer Inst.

[20] MacMahon B, Cole P, Lin TM, Lowe CR, Mirra AP, Ravnihar B, Salber EJ, Valaoras VG, Yuasa S (1970). Age at first birth and breast cancer risk. Bull World Health Organ.

[21] Lambe M, Hsieh CC, Chan HW, Ekbom A, Trichopoulos D, Adami HO (1996). Parity, age at first and last birth, and risk of breast cancer: a population-based study in Sweden. Breast Cancer Res Treat.

[22] Horn J, Opdahl S, Engstrøm MJ, Romundstad PR, Tretli S, Haugen OA, Bofin AM, Vatten LJ, Åsvold BO (2014). Reproductive history and the risk of molecular breast cancer subtypes in a prospective study of Norwegian women. Cancer Causes Control.

[23] Reeves GK, Pirie K, Green J, Bull D, Beral V (2009). Million Women Study Collaborators. Reproductive factors and specific histological types of breast cancer: prospective study and meta-analysis. Br J Cancer.

[24] Lambertini M, Santoro L, Del Mastro L, Nguyen B, Livraghi L, Ugolini D, Peccatori FA, Azim HA (2016). Reproductive behaviors and risk of developing breast cancer according to tumor subtype: A systematic review and meta-analysis of epidemiological studies. Cancer Treat Rev.

[25] Faupel-Badger JM, Arcaro KF, Balkam JJ, Eliassen AH, Hassiotou F, Lebrilla CB, Michels KB, Palmer JR, Schedin P, Stuebe AM, Watson CJ, Sherman ME (2013). Postpartum remodeling, lactation, and breast cancer risk: summary of a National Cancer Institute-sponsored workshop. J Natl Cancer Inst.

[26] Islami F, Liu Y, Jemal A, Zhou J, Weiderpass E, Colditz G, Boffetta P, Weiss M (2015). Breastfeeding and breast cancer risk by receptor status--a systematic review and meta-analysis. Ann Oncol.

[27] Beral V, Bull D, Doll R, Peto R, Reeves G (2004). Collaborative Group on Hormonal Factors in Breast Cancer. Breast cancer and abortion: collaborative reanalysis of data from 53 epidemiological studies, including 83?000 women with breast cancer from 16 countries. Lancet.

[28] Wu JQ, Li YY, Ren JC, Zhao R, Zhou Y, Gao ES (2014). Induced abortion and breast cancer: results from a population-based case control study in China. Asian Pac J Cancer Prev.

[29] Lecarpentier J, Noguès C, Mouret-Fourme E, Gauthier-Villars M, Lasset C, Fricker JP, Caron O, Stoppa-Lyonnet D, Berthet P, Faivre L, Bonadona V, Buecher B, Coupier I (2012). Variation in breast cancer risk associated with factors related to pregnancies according to truncating mutation location, in the French National BRCA1 and BRCA2 mutations carrier cohort (GENEPSO). Breast Cancer Res.

[30] Andrieu N, Goldgar DE, Easton DF, Rookus M, Brohet R, Antoniou AC, Peock S, Evans G, Eccles D, Douglas F, Noguès C, Gauthier-Villars M, Chompret A (2006). Pregnancies, breast-feeding, and breast cancer risk in the International BRCA1/2 Carrier Cohort Study (IBCCS). J Natl Cancer Inst.

[31] Grabrick DM, Hartmann LC, Cerhan JR, Vierkant RA, Therneau TM, Vachon CM, Olson JE, Couch FJ, Anderson KE, Pankratz VS, Sellers TA (2000). Risk of breast cancer with oral contraceptive use in women with a family history of breast cancer. JAMA.

[32] Black MM, Barclay TH, Polednak A, Kwon CS, Leis HP, Pilnik S (1983). Family history, oral contraceptive usage, and breast cancer. Cancer.

[33] Loman N, Johannsson O, Bendahl PO, Borg A, Fernö M, Olsson H (1998). Steroid receptors in hereditary breast carcinomas associated with BRCA1 or BRCA2 mutations or unknown susceptibility genes. Cancer.

[34] Eerola H, Heikkilä P, Tamminen A, Aittomäki K, Blomqvist C, Nevanlinna H Histopathological features of breast tumours in BRCA1, BRCA2 and mutation-negative breast cancer families. Breast Cancer Res.

[35] Tyrer J, Duffy SW, Cuzick J (2004). A breast cancer prediction model incorporating familial and personal risk factors. Stat Med.

[36] Aalen OO (1978). Nonparametric inference for a family of counting processes. Ann Stat.

[37] Cox D (1972). Regression models and life tables. J R Stat Soc Series B.

